# Sensitive detection of *BRAF* V600E mutation by Amplification Refractory Mutation System (ARMS)-PCR

**DOI:** 10.1186/2050-7771-1-3

**Published:** 2013-01-16

**Authors:** Tiangui Huang, Jian Zhuge, Wenyong W Zhang

**Affiliations:** 1 Department of Pathology, Westchester Medical Center and New York Medical College, Valhalla, NY, 10595, USA

## Abstract

**Background:**

*BRAF* mutations occur in approximately 8% of all human cancers and approach 50% in melanoma and papillary carcinoma of thyroid. These mutations provide potentially valuable diagnostic, prognostic and treatment response prediction markers. A sensitive, specific, low-cost assay to detect these mutations is needed.

**Results:**

To detect *BRAF* V600E mutation in formalin-fixed, paraffin-embedded (FFPE) tissue, we developed a method using Amplification Refractory Mutation System (ARMS)-PCR. This method was designed to amplify three products in a single reaction tube: a 200 bp common product serving as an amplification control, a 144 bp *BRAF* V600E specific product, and a 97 bp wild-type (wt) specific product. The sensitivity of this method was determined to be as low as 0.5% for the *BRAF* V600E allele in a wild-type background. This method was successfully validated in 72 thyroid tumors. It detected V600E mutation in 22 out of 33 (67%) of the conventional papillary thyroid carcinoma (PTC), 8 out of 12 (75%) of the tall-cell variant of PTC, whereas none of the 10 follicular variant of PTC showed *BRAF* V600E mutation. In addition, none of the 14 follicular adenomas and 3 follicular carcinomas had *BRAF* V600E mutation. As a comparison method, direct dideoxy sequencing found only 27 out of 30 (90%) mutations detected by ARMS-PCR method, suggesting that this ARMS-PCR method has higher sensitivity.

**Conclusions:**

Our ARMS-PCR method provides a new tool for rapid detection of *BRAF* V600E mutation. Our results indicate that ARMS-PCR is more sensitive than automated dideoxy sequencing in detecting low *BRAF* V600E allele burdens in FFPE tumor specimen. The strategy of this ARMS-PCR design may be adapted for early detection of point mutations of a variety of biomarker genes.

## Background

*BRAF* gene encodes a serine/threonine protein kinase that functions downstream of RAS in the RAS-RAF-MEK-ERK signaling pathway, also known as mitogen activated protein kinase (MAPK) pathway, which is important in regulating cellular responses to extracellular signals including epidermal growth factor (EGF) [1]. Upon activation by receptor binding, RAS proteins recruit RAF to the cellular membrane where RAF is phosphorylated [2]. Activated RAF proteins further phosphorylate MEK1 and MEK2, which then phosphorylate ERK1 and ERK2. Phosphorylated ERK proteins regulate cellular functions through activation of transcription factors including p53, SMAD4, ELK1, c-Myc and c-Fos [2,3]. Activating mutations in RAS and *BRAF* permit constitutive MAPK pathway activation independent of growth factor stimulation, thereby causing deregulation in cellular growth and survival. *BRAF* mutations occur in approximately 8% of all human cancers, with high mutation frequency in malignant melanoma (50-70%), classic papillary carcinoma of the thyroid (40-70%), colorectal cancer (CRC, 5-15%), ovarian cancer and hairy cell leukemia (5-100%) [4,5]. A T1799A transversion resulting in valine-to-glutamate substitution at codon 600 (V600E) accounts for over 80% of all *BRAF* mutations [6,7], and almost all mutations in thyroid tumors [8].

Molecular testing for *BRAF* mutation can provide valuable diagnostic information, treatment selection, and may help predict prognosis. Like *KRAS* mutations, CRC patients with activating *BRAF* mutations do not respond to anti-EGFR monoclonal antibody (MoAb) treatment [9,10]. Because *BRAF* mutations occur in 5-15% of CRC and are usually mutually exclusive with *KRAS* mutations [11], a substantial number of these CRC patients may benefit from *BRAF* mutation testing before initiating MoAb treatment [9]. In thyroid, *BRAF* V600E mutation is restricted to malignant tumors, and is associated with papillary thyroid cancer (PTC), and PTC-derived poorly-differentiated and anaplastic carcinoma [7,8]. Therefore, *BRAF* mutation testing can be helpful as an adjunct method to improve diagnostic accuracy in difficult cases, such as thyroid FNA samples with indeterminate and atypical cytology [12,13].

There are several methods for *BRAF* V600E mutation testing, including dideoxy sequencing, colorimetric Mutector assay, allele-specific real-time PCR, pyrosequencing, high resolution melting (HRM) analysis and COLD-PCR [14-17]. These methods vary in their sensitivity, assay complexity and costs. Automated dideoxy sequencing is considered the “gold standard” method for mutation testing. However, it is limited by relatively complex procedure, overall high costs and low analytical sensitivity of detecting approximately 15-20% mutant allele in a wild-type background [18]. ARMS-PCR is a highly sensitive, specific and low-cost mutation detection method with reported analytical sensitivity ranging from 0.1% to 2% for *JAK2* V617F mutation detection [19,20]. Here we report the development of a highly sensitive and specific ARMS-PCR assay to detect *BRAF* V600E mutation in formalin-fixed, paraffin-embedded (FFPE) tissue with sensitivity as low as 0.5%. We successfully used this assay to characterize the frequency of *BRAF* V600E in thyroid tumors.

## Methods

### Sample source

Cell lines HT-29 (heterozygous for *BRAF* V600E) and K-562 (wt *BRAF* without V600E mutation) were cultured in RPMI1640 supplemented with 10% fetal calf serum. Anonymized FFPE thyroid tumor tissue blocks were obtained from the Department of Pathology for clinical protocol development.

### DNA extraction

3–5 sections of FFPE tissues (5 micron/section) were deparaffinized by xylene and ethanol treatment, and digested with proteinase K at 56°C overnight. Genomic DNA was extracted using ZR genomic DNA I kit (ZYMO Research Corp., Orange, CA) as recommended. DNA extraction from culture cells was otherwise the same as above except no deparaffinization was performed. DNA was then qualified/quantified with ND*-*1000 spectrophotometer (NanoDrop Products, Wilmington, DE).

### ARMS-PCR and sequencing

The ARMS-PCR primer sequences were forward (Fo): 5^′^-CTCTTCATAATGCTTGCTCTGATAG-3′; reverse (Ro): 5′-GCCTCAATTCTTACCATCCAC-3′; forward wild-type identifying (Fiwt): 5′-GTGATTTTGGTCTAGCTACAGT-3′ and reverse mutation identifying (Rimut):5′-CCCACTCCATCGAGATTTC**T**-3′. PCR was performed in a 25 μl final volume containing 1 × Buffer, 2 mM MgCl_2_, 1 unit of Hotstar Taq DNA polymerase (Qiagen Science, Valencia, CA), 200 μM each dNTP, 400 nM primer Fo, 200 nM primer Ro and Fiwt, 800 nM primer Rimut and 30 ng genomic DNA template. PCR amplification was carried out by denaturation at 95°C for 5 min, followed by 40 cycles of 94°C for 20 sec, 68°C for 20 sec and 72°C for 20 sec with a final extension at 72°C for 5 min. PCR products were analyzed by 2% agarose gel electrophoresis. Automated dideoxy sequencing was performed by Macrogen USA (Rockville, MD) after PCR amplification using the Fo-Ro primer pair and the ampliocons purification using QIAquick PCR purification kit (Qiagen).

### Assay sensitivity study

HT-29 cell DNA with heterozygous *BRAF* V600E mutation was serial diluted with DNA from a wt *BRAF* cell line K-562, to generate a sensitivity panel consisting of 0, 0.1%, 0.25%, 0.5%, 1%, 2.5%, 5%, 10%, 20% and 50% of *BRAF* V600E allele in a wild-type background. The PCR was performed under the optimized condition as above.

## Results

### Method establishment

**Figure 1:**
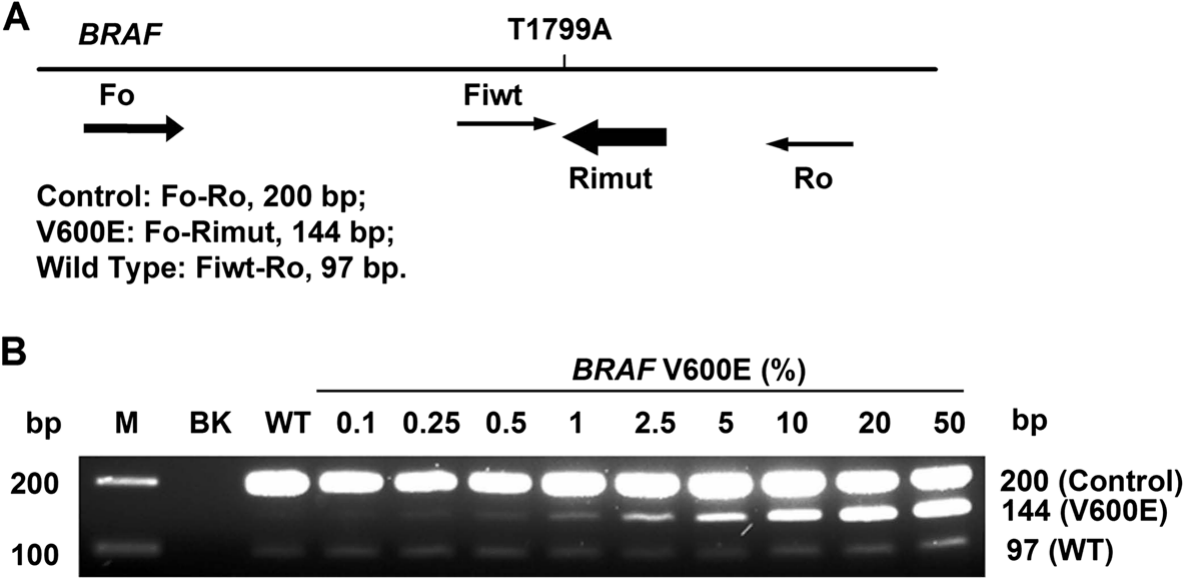
***BRAF *V600E ARMS-PCR.A**, Four PCR primers are included in one PCR tube. Fo-Ro primer pair generates a common fragment of 200 bp flanking the mutation site. Fo-Rimut primer pair generates the 144 bp fragment specific to *BRAF* V600E. Fiwt-Ro primer pair generates a 97 bp fragment specific to wild-type *BRAF*. **B**, Sensitivity study. Agarose gel electrophoresis showed band pattern of a mixing study using *BRAF* V600E positive HT-29 cell DNA spiked into K-562 cell DNA at wide range of ratio namely 0, 0.1%, 0.25%, 0.5%, 1%, 2.5%, 5%, 10%, 20% and 50%. The assay can detect 0.5% *BRAF* V600E allele in a wild-type background. M, 100 bp DNA marker; BK, blank (no DNA) control.

The *BRAF* V600E ARMS-PCR assay contains 4 primers in a single PCR reaction tube with the two outside primers designed to amplify a common fragment of 200 bp flanking the mutation site which can serve as an internal amplification control (Figure 1A). The two internal primers are either specific for the mutant sequence or the wild-type sequence. The mutant and wild-type sequences are distinguishable by the difference of fragment size, as the amplified wild-type fragment is 97 bp, while the mutant fragment is 144 bp. The concentrations of PCR primers and magnesium, the annealing temperature and other cycling parameters were determined by multiple exploring studies, and the condition given here is the final optimized one.

### Assay sensitivity

To assess the analytical sensitivity of the assay, *BRAF* V600E mutation containing DNA was spiked into wild-type *BRAF* DNA, the PCR was performed and showed that the assay we used could detect as low as, if not better than, 0.5% *BRAF* V600E allele in the background of wild-type DNA (Figure 1B). Therefore, the analytical sensitivity of the assay is 0.5%.

### Assay validation and comparison with direct dideoxy sequencing using thyroid tumor specimens

We then analyzed *BRAF* V600E in thyroid tumors using the ARMS-PCR method. A total of 72 thyroid tumors were tested, including 55 PTCs and 17 follicular tumors. Among 55 PTCs, 33 were conventional type, 12 were tall-cell variant, and 10 were follicular variant (Table 1). ARMS-PCR results showed that 22 out of 33 (67%) of the conventional PTC, 8 out of 12 (75%) of the tall-cell variant of PTC, and none of the 10 follicular variant of PTC harbored *BRAF* V600E mutation. Interestingly, none of the 14 follicular adenomas and 3 follicular carcinomas showed *BRAF* V600E mutation.

**Table 1:** *BRAF *V600E mutation rate in thyroid tumors

**Tumor**	**Sample number**	**Positive**	**Positive rate (%)**
**Papillary Thyroid Carcinoma**	**55**	**30**	**55**
Conventional Type	33	22	67
Tall-cell Variant	12	8	75
Follicular Variant	10	0	0
**Follicular Tumor**	**17**	**0**	**0**
Follicular Adenoma	14	0	0
Follicular Carcinoma	3	0	0

**Figure 2:**
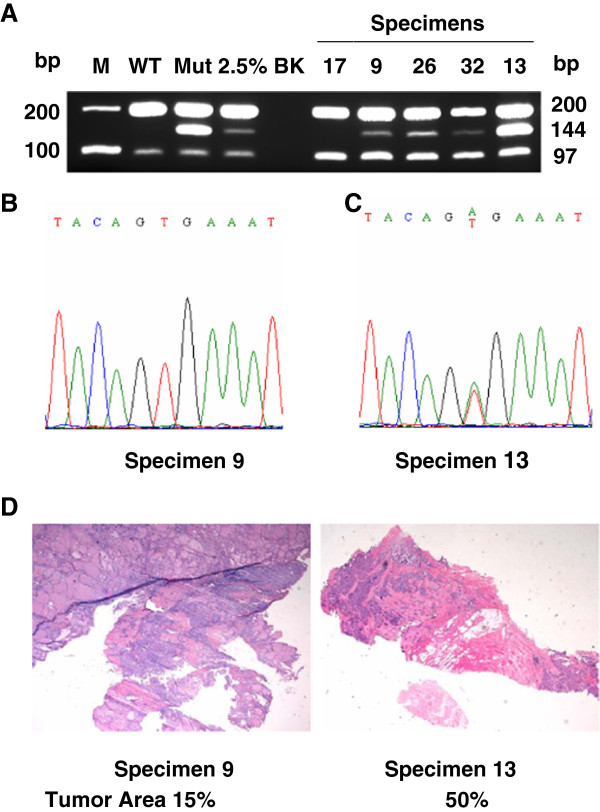
**A, Representative *BRAF *V600E ARMS-PCR results.** Specimen 9, 26 and 32 had a low abundance of *BRAF* V600E mutation with band intensity similar to that of the 2.5% sensitivity control. Specimen 13 was strongly positive for *BRAF* V600E mutation. **B**, Direct dideoxy sequencing of specimen 9 failed to identify *BRAF* V600E mutation as the mutant allele burden was below the limit of detection. **C**, Direct dideoxy sequencing of specimens 13 showed that the mutant allele (A) was more abundant than the wild-type allele (T). **D**, H&E stain of specimens 9 and 13 showed that tumor areas accounted for ~15% and 50% of the total specimen areas, respectively.

As a comparison, automated dideoxy sequencing was used to analyze 42 tumors including all 30 *BRAF* V600E positive tumors and 12 of the *BRAF* V600E negative tumors from ARMS-PCR assayed samples. Among the 30 *BRAF* V600E positive tumors detected with ARMS-PCR assay, direct sequencing identified the mutation in 27 tumors, but failed to identify 3 tumors that had *BRAF* V600E mutation, possibly due to the lower sensitivity of detection of automated dideoxy sequencing. Figure 2A depicts the representative ARMS-PCR results, showing samples 9, 26 and 32 with faint, but clearly visible *BRAF* V600E bands (144 bp). This is comparable to the mutant band intensity of the 2.5% V600E allele burden positive control included in the study. Figure 2B depicts the representative sequencing result for sample 9, showing the mutant signal was below the detection limit of the assay. While ARMS-PCR for sample 13 showed a strong V600E mutant band, and direct sequencing showed A/T double peaks with approximately equal height (Figure 2C). Histologic study of specimen 9 showed that the tumor constituted only a small fraction of the tissue area and sample 13 had a high tumor percentage (Figure 2D). These two cases illustrate the importance of high assay sensitivity of our ARMS-PCR assay to detect low *BRAF* V600E allele burdens in tumor samples. The assay results for the 12 *BRAF* V600E negative tumors were concordant between the ARMS-PCR and direct sequencing. All 14 cases of follicular adenomas had no mutated band detected by ARMS-PCR, showing the specificity of our assay was 100% in this validation study.

Overall, our results indicate that ARMS-PCR is more sensitive than automated dideoxy sequencing in detecting low *BRAF* V600E allele burdens in FFPE tumor specimen.

## Discussion

For the development and implementation of personalized medicine, a sensitive, specific, low-cost and easy-to-implement assay to identify various genetic alterations such as *BRAF* V600E mutation is needed. By selecting primer pair and optimizing PCR cycling conditions, we achieved this goal by using ARMS based PCR. One of the advantages of ARMS-PCR is that the assay is designed to amplify a relative larger common fragment of DNA that flanks the mutation site in all samples regardless of their mutation status. This common fragment conveniently serves as an internal control for template DNA quality as well as potential PCR inhibition. The mutant or wild-type specific PCR amplifications take place in the same reaction tube, thereby allowing the mutant or wild-type specific PCR primers to compete for binding to very limited templates. To increase the sensitivity for detecting this mutation, we tried to change the ratio of four primers and found that the combination of 400 nM primer Fo, 200 nM primer Ro and Fiwt, 800 nM primer Rimut resulted the strongest mutant band. The primer binding to template varies based on their annealing conditions, but relative high Fo and the highest Rimut primer concentrations might favor the production of mutant product in our design. In addition, relative low concentration for Fiwt and Ro primers might reduce full length and wild type product formation by the competition effect of each primer. By such a design, the three products were produced at reasonably comparable levels. Otherwise, the wild type or whole length bands might overshadow the weak mutant band if the mutant *BRAF* V600E abundance is low, which is frequently the case for clinical specimens. With this study, we improved assay sensitivity to as low as 0.5% mutant allele level in the background of wild-type DNA, which is quite sensitive among ARMS-PCR methods [21]. This sensitivity is very difficult to reach with direct dideoxy sequencing which has the sensitivity of 15-20%, and with pyrosequencing which has greatly increased sensitivity to about 2% [22,23]. Considering DNA samples obtained from FFPE tissue is often fragmented due to damage by formalin fixation, we designed our PCR product size to be small (no more than 200 bp) to maximize the chance of successful amplification. This was proven to be helpful since all 72 FFPE samples had successful amplification.

The reported prevalence of *BRAF* V600E mutation for conventional PTC varied from 36% to 67% in the US and European studies [7,8,24-27], and was 83% in a Korean study [28]. The difference in prevalence could be due to different study population or different methodologies used. The prevalence of *BRAF* V600E mutation in the tall-cell variant PTC is in general higher than that of the conventional type, with a mean prevalence of 77% in several studies [8]. On the contrary, the follicular variant of PTC is rarely involved by *BRAF* V600E mutation, with a mean prevalence of 12% [8]. Using ARMS-PCR, we tested 72 thyroid tumors and determined that the frequencies of *BRAF* V600E mutation in the conventional, tall-cell and follicular variants of PTC were 66%, 75% and 0%, respectively. Our frequency of *BRAF* V600E mutation in conventional PTC was at the high end of the reported results, possibly reflecting our improved assay sensitivity compared with automated DNA sequencing, which was the method used in many of the reported studies. Our study showed that DNA sequencing method missed 3 out of 30 PTC samples with *BRAF* V600E detected by ARMS-PCR, resulting in a false negative rate of 10%. In our validation study, the assay specificity is 100% (14/14) based on negative mutation in follicular adenoma. This is consistent with previous reports since benign follicular adenoma has not been found to harbor the *BRAF* mutation [8].

*BRAF* V600E mutation testing has demonstrated utility in helping select CRC patients who are considering monoclonal antibody therapy as wild-type *BRAF* is required for response to anti-EGFR antibodies [9] and improve diagnostic accuracy in thyroid FNA samples [12,13]. In addition, *BRAF* V600E mutation is associated with sporadic microsatellite instable CRC, but not hereditary non-polyposis colorectal cancer (HNPCC) syndrome [29,30]. Therefore, the presence of *BRAF* V600E mutation is an exclusion criteria for HNPCC genetic testing [29,30]. *BRAF* V600E mutation testing can also help facilitate clinical studies of BRAF-targeted therapies [31]. The improved understanding of the role *BRAF* mutations in cancer diagnosis, prognosis and treatment has increased the need for *BRAF* mutation testing [4,32].

New methods are constantly being developed for *BRAF* V600E mutation detection. Shifted termination assay (STA) developed by TrimGen Corporation declared the sensitivity of 1-5% [33]. Lang et al. developed an allele-specific, also known as ARMS, real-time PCR using Taqman probe to increase the sensitivity to 1% and using internal control to exclude false negative results [34]. Morandi et al. developed an allele specific locked nucleic acid (LNA) quantitative PCR assay using LNA-modified allele specific primers and LNA-modified beacon probes to achieve sensitivity of 0.1% [35]. Dual-priming oligonucleotide (DPO)-based multiplex PCR was commercially available from Seegene (Seoul, Korea) and has a sensitivity of 2% [36]. Qiagen developed a *BRAF* mutation detection PCR kit with a sensitivity of 1.27%. Our method using ARMS-PCR therefore appears to have high sensitivity (0.5%) for *BRAF* V600E mutation detection.

## Conclusions

We have developed a sensitive, specific and low-cost ARMS-PCR assay to detect *BRAF* V600E mutation. Comparative study showed that this assay is superior to automated dideoxy sequencing in terms of assay sensitivity, turn-around time and costs. The ARMS-PCR assay can be easily implemented by many molecular laboratories for *BRAF* V600E mutation testing. Our method provides one of the most sensitive methods for *BRAF* V600E gene mutation detection. The principal of our study design can be potentially adapted to detect other low abundance point mutations such as tumors with rich background stroma and post-treatment tumor samples.

## Competing interests

The authors declare that they have no competing interests.

## Authors’ contributions

TH: design, data analysis, paper writing submission. JZ: design experiments and implement. WWZ: design study, sample collection, data analysis and paper writing. All authors read and approved the final manuscript.
